# The descriptive epidemiology of accelerometer-measured physical activity in older adults

**DOI:** 10.1186/s12966-015-0316-z

**Published:** 2016-01-07

**Authors:** K. Berkemeyer, K. Wijndaele, T. White, A. J. M. Cooper, R. Luben, K. Westgate, S. J. Griffin, K. T. Khaw, N. J. Wareham, S. Brage

**Affiliations:** MRC Epidemiology Unit, University of Cambridge, School of Clinical Medicine, Box 285 Institute of Metabolic Science, Cambridge Biomedical Campus, Cambridge, CB2 0QQ UK; Strangeways Research Laboratory, University of Cambridge, Worts Causeway, Cambridge, CB1 8RN UK; Department of Gerontology, University of Cambridge, School of Clinical Medicine, Addenbrooke’s Hospital, Cambridge, CB2 2QQ UK

**Keywords:** Accelerometry, Activity intensity distribution, Older adults, Guidelines

## Abstract

**Background:**

Objectively measured physical activity between older individuals and between populations has been poorly described. We aimed to describe and compare the variation in accelerometry data in older UK (EPIC-Norfolk) and American (NHANES) adults.

**Methods:**

Physical activity was measured by uniaxial accelerometry in 4,052 UK (49–91 years) and 3459 US older adults (49–85 years). We summarized physical activity as volume (average counts/minute), its underlying intensity distribution, and as time spent <100counts/minute, ≥809counts/minute and ≥2020counts/minute both for total activity and that undertaken in ≥10-min bouts.

**Results:**

In EPIC-Norfolk 65 % of wear-time was spent at <100 counts/minute and 20 % spent in the range 100–500 counts/minute. Only 4.1 % of this cohort accumulated more than 30 min/day of activity above 2020 counts/minute in 10-min bouts. If a cut-point of >809 counts/minute is used 18.7 % of people reached the 30 min/day threshold. By comparison, 2.5 % and 9.5 % of American older adults accumulated activity at these levels, respectively.

**Conclusion:**

As assessed by objectively measured physical activity, the majority of older adults in this UK study did not meet current activity guidelines. Older adults in the UK were more active overall, but also spent more time being sedentary than US adults.

**Electronic supplementary material:**

The online version of this article (doi:10.1186/s12966-015-0316-z) contains supplementary material, which is available to authorized users.

## Background

Physical activity changes throughout the life-course, especially in the transition to older adulthood [1]. Given the importance of physical activity for the primary and secondary prevention of major chronic diseases and maintenance of independence in older age [2], it is important to understand the levels and patterns of physical activity in this age group.

The majority of population-based studies have used self-report methods to assess physical activity, which have a number of limitations including problems of recall and reporting bias [3]. These limitations may be exacerbated in older populations, in whom cognitive impairment is more likely [4]. In addition, due to the ubiquitous nature of low intensity activities and sedentary behaviors, these tend to be particularly difficult to recall [5]; however, such behaviors may be of particular importance in older adults as low intensity activities are more prevalent in this age group [1]. Objective physical activity assessment methods, such as accelerometry, enable more accurate assessment of the entire movement intensity spectrum and may therefore have particular merits in older populations.

There are, however, challenges in interpreting data from objective physical activity measurement methods. Several of these arise from classifying activity into broad intensity categories, often labeled as ‘sedentary, light, moderate, and vigorous’, rather than using the full continuous distribution of intensity. For accelerometry data, the cut-points used to define these classical intensity categories are typically based on calibration studies using energy expenditure as a criterion method during specific lab-based activities, which may or may not apply to all age groups and populations [6].

Physical activity guidelines suggest that older adults should be ‘as active as they can’ while aiming to achieve the recommended physical activity levels for adults, i.e., 30 min of moderate-to-vigorous physical activity (MVPA) per day in bouts of ≥10 min [2, 7]. The evidence base for the bout requirement, however, is limited and it is also uncertain how to appropriately define intensity, especially in an older age group [5] in whom there is large inter-individual variation in the intensity associated with a given activity. Therefore, a single cut-point above which an activity might be regarded as ‘moderate’ for older adults is difficult to establish, which explains the diversity of cut-points used to define MVPA [8–10].

Activities of at least moderate intensity have been the main focus of several studies objectively describing physical activity in older adults [3, 8, 11], but the focus on this specific cut-point may disregard a whole range of relevant activities of lesser intensity that could be important contributors to overall physical health and well-being [12]. While a number of studies have described time spent at different activity intensities in older adulthood [3, 13–16], to our knowledge the continuous distribution of activity intensity in this age group, using objective activity assessment, has only been described in the British 1946 birth cohort, showing that activity time is inversely associated with intensity [17].

Another area of uncertainty is how populations from different countries compare in terms of key activity characteristics. International comparisons of physical activity levels may point to certain determinants of physical activity and are essential to guide global public health promotion. So far, however, only a few studies in adults have used objective accelerometry data and implemented the same processing procedures to maximize comparability [9, 18].

Therefore, the main aim of this study was to provide a description of objectively measured physical activity in older UK adults, both in terms of overall physical activity volume and time distribution across the entire physical activity intensity spectrum. We also compared these estimates to US adults of a similar age from the NHANES study using the same accelerometry processing procedures in both cohorts.

## Methods

### Participants and protocol

#### EPIC-Norfolk

The Norfolk arm of the European Prospective Investigation of Cancer (EPIC-Norfolk) has been described in detail elsewhere [19]. In brief, 25,639 men and women aged 40–79 years, who were recruited from 35 general practices in the area of Norfolk, consented to participate in the EPIC-Norfolk study and attended a first health check between 1993–1997. A 2^nd^ health check was conducted 1997–2000 at which physical activity was measured by self-report only. Between 2006 and 2011, 8,623 men and women participated in the 3rd health check and a subsample of 4,148 were asked to wear an accelerometer (ActiGraph model GT1M, ActiGraph, Pensacola, FL) for the assessment of free-living physical activity [19].

At the 3rd health check visit, height and weight were measured using standard anthropometric techniques. Employment status, education level and self-rated health were assessed by self-report questionnaires. At the end of the clinical visit, participants were asked to wear the accelerometer on a belt around the right hip during all waking hours for 7 days, and to only take it off for water-based activities (e.g., when showering) and during sleep. Accelerometers were set up to capture data in 5-s epochs.

#### NHANES

To compare objectively measured physical activity levels between UK and American older adult populations, a subsample of 3459 participants of the NHANES 2003–2004 cohort was used, excluding participants aged <49 years (the lowest age in the EPIC-Norfolk 3^rd^ health check). NHANES is a national population-based study of the civilian, non-institutionalized US population aged ≥6 years, as previously described in detail [10, 20]. As in the EPIC-Norfolk protocol, NHANES 2003–2004 participants were asked to wear an accelerometer (ActiGraph AM7164) for 7 consecutive days on a belt around the hip, initialized to capture data in 60-s epochs. Accelerometer records deemed to be out-of-calibration or unreliable by the NHANES team were excluded.

### Accelerometry data processing

To maximize comparability between studies, we processed the accelerometry data of both studies using the same criteria, after first collapsing the 5-s EPIC-Norfolk data to 60-s epoch resolution. The two models of ActiGraph use either a piezo-electric (AM7164) or a MEMS-based (GT1M) acceleration sensor, both of which have a frequency-dependent response to acceleration magnitude [21, 22]. Both have been shown to be approximately comparable in the human movement range, except at the extreme low end [23]; this necessitates a slight revision of the wear/non-wear classification compared to what has been used elsewhere for AM7164 data only [10]. Continuous zero strings of ≥90 min were defined as non-wear time, which is similar to other studies using MEMS-based Actigraph versions [8, 24]; for this age-group this threshold resulted in a more realistic pattern of 2–4 wear/ non-wear transitions per day, compared to using zero strings ≥60 min (see example in Additional file 1: Figure S1). One would expect at least two transitions from getting up (non-wear to wear) and going to bed (wear to non-wear). A valid day was defined as ≥600 min of wear time [25]. Only participants with ≥4 valid days of data were included in analyses. As more than 200 participants (mainly in the NHANES) were found to have acceleration data for longer than 19 h/day (indicating monitor wear during sleep), we truncated wear-time to 19 h/day and time spent in the <100 counts/minute intensity category for both cohorts to normalize the data.

We derived overall physical activity volume, defined as total counts divided by wear time, as well as the movement intensity distribution as a continuous construct in 15 systematic intervals. In addition, we report time spent <100 counts/minute, as well as time spent above the commonly used MVPA cut-points of 2020 counts/minute [10] and 809 counts/minute [23]. The latter cut-point was identified as the lower boundary for MVPA in a validation study of 20 older adults (aged 60–90 years) performing treadmill-based and free-living walking activities around 3 METs [26]. To indicate the degree of activity accumulation occurring in bouts, we also analyzed time spent ≥2020 and ≥809 counts/minute in continuous bouts lasting ≥10 min.

### Statistical analysis

Due to the non-normal distribution of physical activity, most descriptive characteristics are described using medians and interquartile ranges (IQR) and some using means and standard deviation (i.e., age). Univariate non-parametric tests for trend were used to examine differences in physical activity by age group, employment status, education level, BMI, and self-rated health, in men and women separately. We also examined the robustness of these differences by mutually adjusting for all stratifiers. Stata (StataCorp LP) version 13.1 was used for all analyses.

## Results

### EPIC-Norfolk

A total of 4,052 EPIC-Norfolk participants had ≥4 valid days (>10 h/day) of accelerometer data and were included in analyses (98.0 % of the whole accelerometry sample, Table 1). Mean accelerometer wear-time was 14.5 h/day (SD 1.1 h/day) and was slightly higher in men than women (14.7 vs 14.4 h/day, *P* < 0.05). Participants with valid accelerometer data did not differ significantly from those who did not wear an accelerometer with respect to age, sex, BMI, education level and self-rated health (Additional file 2: Table S1).

**Table 1:** Participant Characteristics for the EPIC-Norfolk Accelerometry Sample by Gender (3rd health check, 2006–2011)

		All	Men	Women
		n = 4052	n = 1797 (44.3 %)	n = 2255 (55.7 %)
Age, *mean (SD)*		69.0 (7.6)	70.0 (7.6)	68.2 (7.5)
Paid employment	yes % (n)	25.8 (1028)	29.2 (518)	23.1 (510)
	no % (n)	74.3 (2957)	70.8 (1255)	76.9 (1678)
Education level	O-level or lower % (n)	37.3 (1512)	30.5 (548)	42.8 (964)
	A-level % (n)	45.3 (1835)	49.7 (893)	41.8 (942)
	Degree % (n)	17.4 (704)	19.8 (355)	15.5 (349)
BMI	underweight % (n)	0.7 (28)	0.4 (7)	0.9 (21)
	normal weight % (n)	34.9 (1414)	27.2 (489)	41.0 (925)
	overweight % (n)	45.8 (1855)	54.4 (978)	38.9 (877)
	obese % (n)	18.6 (755)	18.0 (323)	19.2 (432)
Smoking	current % (n)	4.0 (159)	3.9 (69)	4.1 (90)
	former/never % (n)	96.0 (3829)	96.1 (1702)	96.0 (2127)
Self-rated health	very good/excellent % (n)	43.1 (1706)	42.6 (748)	43.5 (958)
	good % (n)	42.2 (1671)	41.8 (734)	42.6 (937)
	fair/poor % (n)	14.7 (581)	15.7 (275)	13.9 (306)

Absolute numbers do not add up to the column total due to missing data

Total physical activity volume (average movement intensity, counts/minute) did not differ between men and women (254.8 vs 254.0, p = 0.8). As shown in Table 2, physical activity volume was significantly lower across increasing strata of age and BMI and across decreasing strata for self-rated health and education, in both men and women. Physical activity volume was also significantly higher in those who reported paid employment compared to those who were not employed or were retired. A comparable pattern of association with all stratifiers was found in both sexes for time spent ≥809 and ≥2020 counts/minute, irrespective of whether this was accumulated in bouts of ≥10 min or not. Time spent <100 counts/minute followed the opposite pattern for age, BMI and self-reported health, showing increasingly higher levels of sedentary time across increasing strata for age and BMI and across decreasing strata for self-reported health. Participants who reported being employed spent less time sedentary compared to those not employed. The association between sedentary time and education level was less strong, and not significant in women. Mutual adjustment for stratifiers indicated that the strongest factors associated with physical activity volume and time spent at the lowest (<100 counts/minute) and highest physical activity intensities (≥809 or ≥2020 counts/minute) were age, BMI and self-rated health (*P* < 0.05).

**Table 2:** Physical Activity by Socio-demographic Factors in EPIC-Norfolk Men and Women

		Overall PA volume	Time below 100 cpm	Time above 809 cpm		Time above 2020 cpm	
		Counts per minute	min/day	min/day, not considering bouts	min/day in bouts of at least 10 min	min/day, not considering bouts	min/day in bouts of at least 10 min
		median (IQR)	median (IQR)	median (IQR)	median (IQR)	median (IQR)	median (IQR)
Men n = 1797 (44.3 %)							
Age	<60 years	305 (234, 389)	584 (531, 645)	96 (74, 129)	17 (7, 33)	28 (17, 44)	5 (0, 14)
	60-70 years	276 (204, 364)	576 (518, 627)	88 (60, 122)	16 (6, 33)	25 (13, 39)	3 (0, 13)
	70-80 years	216 (155, 288)	602 (544, 643)	64 (41, 95)	12 (3, 24)	14 (6, 29)	1 (0, 8)
	>80 years	120 (80, 178)^**^	643 (584, 697)^**^	29 (13, 52)^**^	2 (0, 10)^**^	3 (1, 11)^**^	0 (0, 1)^**^
Paid employment	yes	287 (219, 368)	577 (523, 630)	94 (68, 128)	14 (6, 29)	24 (13, 39)	3 (0, 10)
	no	215 (150, 304)^**^	599 (543, 649)^**^	65 (38, 97)^**^	11 (2, 27)^**^	16 (5, 31)^**^	2 (0, 10)
Educational level	O-level & lower	227 (156, 309)	591 (536, 648)	72 (42, 102)	11 (3, 25)	16 (6, 31)	1 (0, 7)
	A-level	240 (165, 327)	586 (530, 639)	73 (44, 110)	12 (3, 27)	18 (7, 33)	2 (0, 9)
	Degree	255 (179, 339)^*^	605 (556, 653)^**^	77 (52, 109)	17 (6, 32)^**^	24 (12, 40)^**^	5 (0, 15)^**^
BMI	normal weight	277 (201, 361)	576 (419, 626)	86 (56, 119)	18 (5, 33)	25 (11, 41)	4 (0, 15)
	overweight	237 (168, 325)	591 (537, 644)	75 (45, 106)	12 (4, 27)	19 (8, 34)	2 (0, 9)
	obese	181 (121, 261)^**^	618 (562, 669)^**^	52 (31, 81)^**^	5 (0, 18)^**^	10 (3, 23)^**^	0 (0, 5)^**^
Smoking	current	218 (120, 321)	596 (524, 669)	67 (29, 110)	6 (0, 21)	12 (3, 27)	0 (0, 2)
	former/never	238 (167, 327)	592 (536, 644)	74 (47, 107)	13 (4, 28)	19 (8, 34)	2 (0, 10)
Self-rated health	very good	267 (201, 359)	580 (528, 632)	88 (59, 121)	18 (6, 33)	24 (11, 38)	3 (0, 13)
	good	230 (163, 309)	596 (541, 644)	70 (44, 102)	12 (3, 26)	18 (8, 32)	2 (0, 10)
	poor	169 (110, 243)^**^	620 (561, 684)^**^	45 (24, 79)^**^	5 (0, 15)^**^	7 (2, 21)^**^	0 (0, 4)^**^
Women n = 2255 (55.7 %)							
Age	<60 years	274 (217, 351)	581 (491, 581)	86 (63, 116)	13 (4, 26)	22 (10, 34)	4 (0, 13)
	60-70 years	267 (207, 341)	530 (480, 582)	83 (58, 113)	14 (4, 26)	19 (9, 32)	3 (0, 11)
	70-80 years	210 (146, 271)	562 (507, 617)	57 (33, 88)	6 (1, 16)	10 (3, 19)	0 (0, 5)
	>80 years	134 (97, 190)^**^	612 (562, 670)^**^	28 (19, 51)^**^	2 (0, 7)^**^	3 (1, 8)^**^	0 (0, 0)^**^
Paid employment	yes	274 (212, 345)	536 (487, 583)	86 (60, 116)	12 (4, 25)	20 (10, 33)	3 (0, 11)
	no	228 (166, 306)^**^	551 (491, 606)^**^	68 (41, 97)^**^	9 (2, 22)^**^	13 (5, 26)^**^	1 (0, 8)^**^
Educational level	O-level & lower	227 (164, 303)	548 (495, 602)	66 (41, 98)	8 (2, 20)	12 (5, 24)	0 (0, 6)
	A-level	241 (182, 317)	545 (489, 601)	74 (47, 104)	10 (3, 22)	15 (7, 28)	2 (0, 8)
	Degree	267 (195, 344)^**^	551 (495, 601)	81 (55, 111)^**^	15 (5, 27)^**^	20 (9, 33)^**^	4 (0, 13)^**^
BMI	normal weight	271 (204, 344)	532 (481, 583)	83 (56, 112)	14 (4, 26)	19 (9, 33)	3 (0, 11)
	overweight	232 (167, 307)	551 (500, 603)	67 (42, 100)	9 (2, 20)	14 (6, 26)	1 (0, 8)
	obese	205 (139, 263)^**^	572 (510, 630)^**^	60 (32, 86)^**^	5 (0, 14)^**^	7 (2, 17)^**^	0 (0, 2)^**^
Smoking	current	214 (174, 299)	546 (484, 597)	64 (43, 97)	6 (1, 18)	11 (5, 25)	0 (0, 5)
	former/never	240 (177, 317)	547 (493, 602)	72 (46, 103)	10 (3. 23)	15 (6, 28)	2 (0, 9)
Self-rated health	very good	267 (206, 341)	538 (485, 588)	83 (57, 112)	14 (5, 26)	19 (10, 32)	3 (0, 12)
	good	228 (171, 301)	551 (494, 605)	67 (43, 97)	8 (2, 20)	13 (5, 24)	1 (0, 7)
	poor	188 (128, 255)^**^	563 (513, 631)^**^	51 (25, 80)^**^	3 (0, 16)^**^	6 (2, 16)^**^	0 (0, 2)^**^

*Data are medians and interquartile ranges. *P*-values denote significance of non-parametric test for trend across categories (^*^ *P* < 0.05, ^**^ *P* < 0.01). Values are rounded

**Fig. 1:**
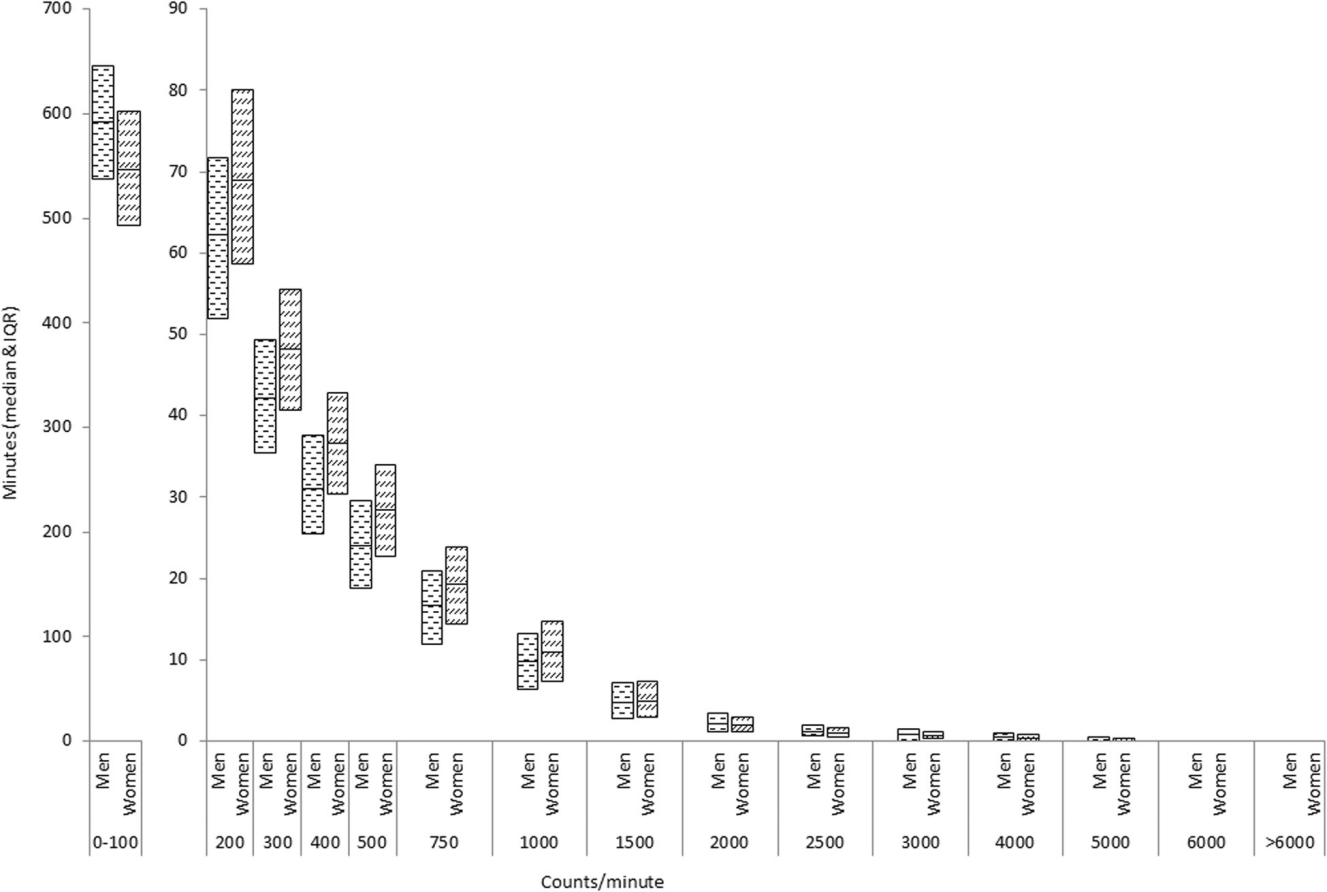
Distribution of Movement Intensity in EPIC-Norfolk Men and Women (Median and IQR, normalized to 100-counts/minute width)

The movement intensity distribution, expressed in 15 intervals of 100 counts/minute width, is presented in Fig. 1. As expected, the distribution is positively skewed with the vast majority of activity occurring in the lower intensity ranges. Most recorded time (64.5 %) was spent being sedentary (<100 counts/minute). The majority of the remaining non-sedentary time (18.2 % in men, 21.2 % in women) was of very light intensity between 100 and 500 counts/minute. Only about 2.7 % of all activity in men and 2.2 % of all activity in women occurred above the cut-point of 2000 counts/minute. There were differences between men and women for activity intensities. Men spent 44 min/day longer sedentary whereas women spent more time in all categories up to 2000 counts/minute. However, men spent more time than women with activity greater than 2000 counts/minute (*P* < 0.001).

### Comparison between EPIC-Norfolk and NHANES

A total of 3459 NHANES participants met the accelerometry inclusion criteria and were compared to the EPIC Norfolk sample. Mean (SD) age of included NHANES participants was 66.4 (10.1) years and 50.7 % were men, comparable to the EPIC-Norfolk cohort (Table 1). Weight status was comparable between the two populations, with 72 % and 64 % of participants being overweight or obese in NHANES and EPIC-Norfolk, respectively. In terms of education, 31.1 % of the NHANES participants had less than a High School degree (24.7 % High School graduates), whereas 37.3 % of EPIC-Norfolk participants were educated to less than A-level (45.3 % A-level graduates).

**Fig. 2:**
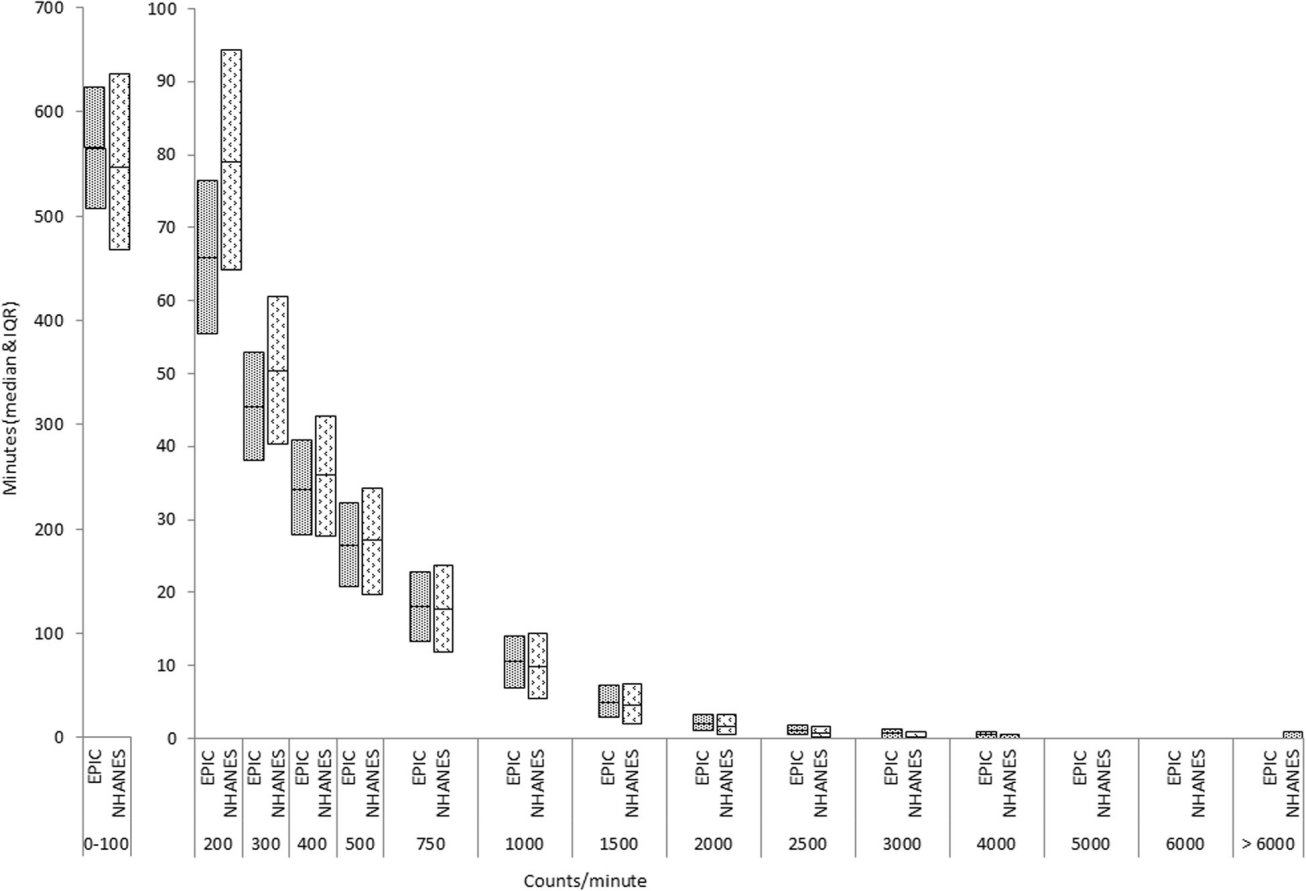
Distribution of Movement Intensity in EPIC-Norfolk and NHANES (Median and IQR, normalized to 100-counts/minute width)

Monitor wear-time did not differ between the two studies (EPIC-Norfolk: 872 min/day, NHANES: 871 min/day, *P* < 0.05). Activity volume was higher in the UK sample compared to the US (238.2 vs 205.2 counts/minute, *P* < 0.05). As shown in Fig. 2, EPIC-Norfolk participants spent 13 min/day more being sedentary than NHANES participants (566.3 min/day vs 553.4 min/day, *P* < 0.05), NHANES participants spent more time in the lower intensity categories than EPIC-Norfolk participants. EPIC-Norfolk participants spent more time than those in NHANES undertaking physical activity >750 counts/minute.

The adherence to physical activity guidelines for older adults, as determined by ≥30 min/day of movement intensity ≥2020 counts/minute undertaken in bouts of ≥10 min, was low in both cohorts, with 4.1 % of all participants in EPIC-Norfolk and 2.5 % in NHANES accumulating activity at this level. Using the cut-point of ≥809 counts/minute, the adherence level was 18.7 % in EPIC-Norfolk and 9.5 % in NHANES. When counting all activity ≥2020 counts/minute whether or not it was undertaken in bouts lasting more than 10 min, 26.7 % of EPIC-Norfolk participants and 13.9 % of NHANES participants spent ≥30 min/day in this intensity spectrum. The equivalent numbers for the ≥809 counts/minute definition were 86.8 % (EPIC-Norfolk) and 73.4 % (NHANES).

**Fig. 3:**
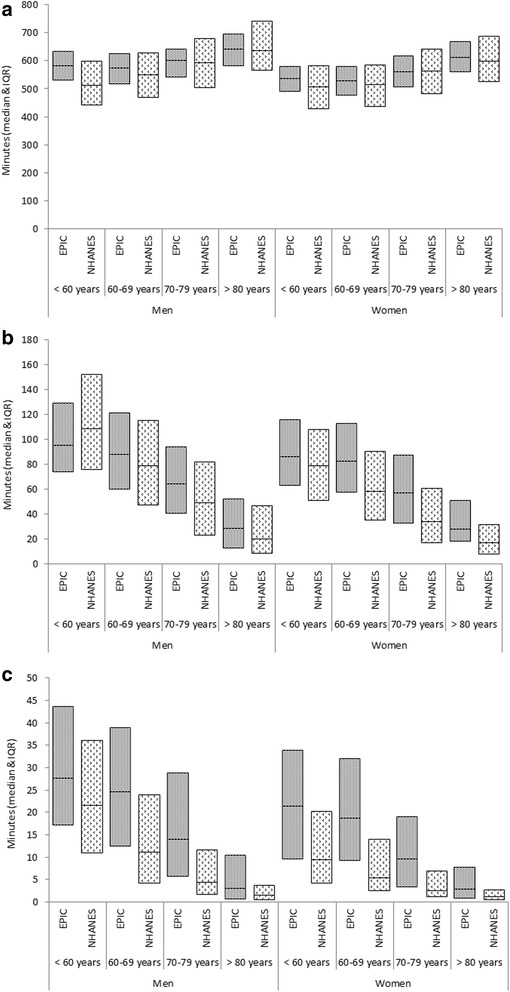
Median and IQR of Average Daily Physical Activity between 0 and 100 counts/minute, ≥809 counts/minute and ≥2020 counts/minute, stratified by Gender, Age Category and Cohort. **a**. Time between 0 and 100 counts/minute (adjusted for non-normal awake wear time). **b**. Time above 809 counts/minute. **c**. Time above 2020 counts/minute

Figures 3a-c show the minutes of activity spent <100, ≥809 and ≥2020 counts/minute for EPIC-Norfolk and NHANES participants, stratified by sex and age group. Overall, sedentary time (Fig. 3a) was lower in women than in men in both cohorts. In EPIC-Norfolk, men and women below the age of 60 years and between 60 and 70 years of age spent less time sedentary compared to the older age groups. However, overall the differences between age groups were greater in NHANES than in EPIC-Norfolk participants. The population variance of sedentary time was larger in the NHANES study. EPIC-Norfolk men and women of all ages generally spent more time above 809 and 2020 counts/minute than the NHANES participants (Fig. 3b & c) with the exception of men in the youngest age group in NHANES.

## Discussion

The aim of this study was to provide a detailed description of objectively measured physical activity in older adults by socio-demographic characteristics, BMI and self-rated health, as well as to directly compare similarly aged older adults from the UK and the US. We have shown that the continuous physical activity intensity distribution is patterned differently, both within and between populations. Women from the EPIC-Norfolk study accumulate their overall activity through activities of lower intensities while men accumulate a similar activity volume by spending more time sedentary as well as in activity intensities above 2000 counts/minute. When examining differences by age, we found a stronger decline in higher intensity activity when comparing UK participants aged <70 years with those aged >70 years, which was accompanied by a more pronounced increase in sedentary time. BMI and self-rated health appeared as additional important correlates. In comparison with the US, the UK older adults generally performed more activity of higher intensity (≥809 and ≥2020 counts/minute), and showed a less steep decline in higher intensity activity across age groups, especially for activity ≥809 counts/minute. The EPIC-Norfolk population also displayed a less steep increase in sedentary time across increasing age groups, which was driven in part by the younger participants having greater sedentary time than in NHANES.

Recently, Jefferis et al. [8] demonstrated that 10 % and 15 % of older British women and men >70 years accumulated the recommended level of ≥30 min/day of MVPA in ≥10 min bouts, using an accelerometer threshold of >1040 counts/minute, which was reduced to 3 % and 7 % respectively when they used a threshold of >1952 counts/minute. These estimates are comparable to our prevalence estimates in EPIC-Norfolk 16.1 % in women and 22.2 % in men for the ≥809 counts/minute cut-point and 3.2 % in women and 5.4 % in men for the ≥2020 counts/minute cut-point, respectively when using a 10-min minimal bout duration criterion. Compared with Swedish adults aged 60–75 years old who spent on average 29 (men) or 23 (women) minutes/day in activity intensity ≥2020 counts/minute, EPIC-Norfolk men and women accumulated less time in this intensity category; 23.6 and 18.9 min/day, respectively. Furthermore, in Swedish older adults, around 55 % of the time spent ≥2020 counts/minute was accumulated in bouts longer than 10 min, whereas in EPIC-Norfolk participants only around 31 % of time ≥2020 counts/minute was spent in 10-min bouts [9]. Different processing criteria obviously result in stark differences in prevalence estimates when considering all MVPA time, regardless of occurrence in bouts, e.g., 19 % and 56 % of women accumulated ≥30 min/day of MVPA at the higher and lower MVPA cut-point in the study by Jefferis et al., with corresponding numbers in men being 27 % and 62 % [8]. This concept of time spent in MVPA regardless of durations of the bouts was also examined in the British 1946 birth cohort of older adults in 2006–2010, in which 43 % of women and 60 % of men accumulated over 30 min/day above 3 METs, as estimated by combined heart rate and movement sensing [17]. The equivalent percentage of the population spending more than 30 min/day above the cut-points used in EPIC-Norfolk (≥809 and ≥2020 counts/minute) were 86.8 % and 26.7 %, respectively.

The differences that we have observed between strata and studies for achievement of physical activity targets raise the question about whether a cut-point of 2020 counts/minute is appropriate to characterize “moderate” activity as defined in physical activity guidelines. Differences in the degree of adherence to recommendations between men and women or between age groups can be explained by variation in how activity time is spent. However, a number of studies have demonstrated that there are health benefits of physical activity in the light intensity range below 2020 counts/minute [27]. It is currently difficult to define a lower boundary for the minimum amount of activity that is beneficial for health. In the future, revision of guidelines will need to be based on epidemiological data, which includes the full range of movement intensity, rather than data that have been condensed into broad categories. This is a particular issue in older participants for whom activity of lower intensity may be especially important.

This study has several strengths. Firstly, it provides a high-resolution description of the full intensity distribution of physical activity, as well as a detailed description of higher intensity physical activity, the accumulation of bouts of physical activity at two different levels of intensity and sedentary time in EPIC-Norfolk and NHANES. Secondly, with more than 4000 participants, the EPIC-Norfolk cohort is the largest cohort in UK adults in which physical activity was measured by hip accelerometry. Finally, implementation of the same processing procedures allowed a direct comparison between the two cohorts.

There are also limitations of this study that need to be considered when interpreting results. At baseline, EPIC-Norfolk participants were found to be representative of the Health Survey for England population for age and sex as well as all measured anthropometric characteristics [28]. However, participants who returned to the 3^rd^ health check were, on average, younger and had a healthier cardiovascular risk profile and higher socio-economic status than the baseline population, although the sample still represents a variety of socio-economic backgrounds and lifestyle factors [19]. A comparison of basic socio-demographic characteristics between participants with and without accelerometry at the 3^rd^ health check did not show any difference between the main socio-demographic and health characteristics. Furthermore, as this was the first time point at which physical activity was measured objectively in this cohort, the data are cross-sectional and therefore yield no information on within-individual change of activity with age. It is possible that not wearing the monitor during sleep or water-based activities, and the use of an automated non-wear algorithm may have resulted in some misclassification between non-wear and sedentary time. In addition, we have collapsed the raw EPIC data in 5-s resolution to 60-s resolution for the purpose of comparison to other population data; intensity estimates using 5-s data are likely to differ [25, 29]. The GT1M model of the accelerometer used in EPIC-Norfolk is less sensitive at the very low end of the movement continuum than the model used in NHANES, leading to potential differential properties of the zero-strings used to classify non-wear [13, 14], which additionally supports our decision to relax the zero-string criteria for defining non-wear time from 60 to 90 min as this produced similar wear time estimates between cohorts. Non-normally long wear-times were found, mainly in the NHANES dataset, leading to a possible overestimation of wear-time and time spent in the lowest intensity category. Therefore, sedentary time was normalized for participants with a wear-time >19 h/day in both cohorts. Finally, activities which are less well detected by hip-accelerometry (e.g., cycling) may have a different prevalence between UK and US older adults, in this way potentially biasing our comparison for overall physical activity volume and higher intensity activity between the cohorts [30].

## Conclusion

The semi-continuous distribution of movement intensity showed that UK women spent significantly more time in lower intensity physical activity categories whereas men spent more time being sedentary and in high intensity physical activity categories, while overall physical activity volume was not significantly different between sexes. As a large proportion of time is spent in the light intensity range, the entire movement distribution should be considered when analyzing physical activity as it is possible that activities of this intensity may also confer health benefits in addition to those already established for higher intensity. Finally, this study also showed that there are differences between UK and US older adults, with the latter having lower overall activity volume and less time in higher intensity physical activity, as well as a steeper age gradient in time spent in higher intensity physical activity. However, and maybe contrary to expectations, American older adults spent less time being sedentary. This suggests that effective physical activity promotion in older adults should specifically aim to delay the decline of physical activity with age. With this in mind, consideration of the whole intensity distribution may aid our ability to detect subtle but important effects of physical activity promotion in the globally growing population of older people.

## Additional files

Additional file 1: Figure S1. Example accelerometer file showing time segments classified as non-wear using the 90-min and the 60-min zero string criterions. (JPG 80 kb) Additional file 2: Table S1. Differences between participants with and without accelerometry at 3rd health check, EPIC-Norfolk. (XLSX 17 kb)

## Abbreviations

BMI: Body Mass Index
EPIC: European Prospective Investigation into Cancer
MET: Metabolic equivalent of task
MVPA: Moderate-to-vigorous physical activity
NHANES: National Health and Nutrition Examination Survey

## References

[1] Aresu M, Bécares L, Brage S, Chaudhury M, Doyle-Francis M, Esliger D, et al. Health Survey for England 2008: Volume 1 Physical activity and fitness. NHS, The Information Centre for Health and Social Care. 2011. p. 894–910. Available from: http://www.hscic.gov.uk/pubs/hse08physicalactivity.

[2] Department of Health. Start Active, Stay Active: A report on physical activity for health from the four home countries’ Chief Medical Officers. Strategy. 2011. https://www.gov.uk/government/publications/start-active-stay-active-a-report-on-physical-activity-from-thefour-home-countries-chief-medical-officers.

[3] Sun F, Norman IJ, While AE (2013). Physical activity in older people: a systematic review. BMC Public Health.

[4] Rait G, Fletcher A, Smeeth L, Brayne C, Stirling S, Nunes M (2005). Prevalence of cognitive impairment: Results from the MRC trial of assessment and management of older people in the community. Age Ageing.

[5] Wareham NJ, Rennie KL (1998). The assessment of physical activity in individuals and populations: why try to be more precise about how physical activity is assessed?. Int J Obes Relat Metab Disord.

[6] Janz KF (2006). Physical activity in epidemiology: moving from questionnaire to objective measurement. Br J Sports Med.

[7] NHS Choices. Factsheet 5: Physical activity guidelines for Older Adults (65+ years). NHS Choices; 2013. Available from: http://www.hscic.gov.uk/pubs/hse08physicalactivity.

[8] Jefferis BJ, Sartini C, Lee I-M, Choi M, Amuzu A, Gutierrez C (2014). Adherence to physical activity guidelines in older adults, using objectively measured physical activity in a population-based study. BMC Public Health.

[9] Hagströmer M, Troiano RP, Sjöström M, Berrigan D (2010). Levels and patterns of objectively assessed physical activity-a comparison between Sweden and the United States. Am J Epidemiol.

[10] Troiano RP, Berrigan D, Dodd KW, Mâsse LC, Tilert T, Mcdowell M (2008). Physical activity in the United States measured by accelerometer. Med Sci Sports Exerc.

[11] Tucker JM, Welk GJ, Beyler NK (2011). Physical activity in U.S. adults: Compliance with the physical activity guidelines for Americans. Am J Prev Med.

[12] Buman MP, Hekler EB, Haskell WL, Pruitt L, Conway TL, Cain KL (2010). Objective light-intensity physical activity associations with rated health in older adults. Am J Epidemiol.

[13] Lohne-Seiler H, Hansen BH, Kolle E, Anderssen S a (2014). Accelerometer-determined physical activity and self-reported health in a population of older adults (65–85 years): a cross-sectional study. BMC Public Health.

[14] Evenson KR, Buchner DM, Morland KB (2012). Objective measurement of physical activity and sedentary behavior among US adults aged 60 years or older. Prev Chronic Dis.

[15] Copeland JL, Esliger DW (2009). Accelerometer assessment of physical activity in active, healthy older adults. J Aging Phys Act.

[16] Davis MG, Fox KR (2007). Physical activity patterns assessed by accelerometry in older people. Eur J Appl Physiol.

[17] Golubic R, Martin KR, Ekelund U, Hardy R, Kuh D, Wareham N (2014). Levels of physical activity among a nationally representative sample of people in early old age: results of objective and self-reported assessments. Int J Behav Nutr Phys Act.

[18] Corder K, Van Sluijs EMF (2010). Invited commentary: Comparing physical activity across countries-current strengths and weaknesses. Am J Epidemiol.

[19] Hayat S a, Luben R, Keevil VL, Moore S, Dalzell N, Bhaniani A (2014). Cohort profile: A prospective cohort study of objective physical and cognitive capability and visual health in an ageing population of men and women in Norfolk (EPIC-Norfolk 3). Int J Epidemiol.

[20] Atienza A a, Moser RP, Perna F, Dodd K, Ballard-Barbash R, Troiano RP (2011). Self-reported and objectively measured activity related to biomarkers using NHANES. Med Sci Sports Exerc.

[21] Brage S, Brage N, Wedderkopp N, Froberg K (2003). Reliability and Validity of the Computer Science and Applications Accelerometer Model 7164 in a Mechanical Setting. Meas Phys Educ Exerc Sci.

[22] Rothney MP, Apker GA, Song Y, Chen KY (2008). Comparing the performance of three generations of ActiGraph accelerometers. J Appl Physiol.

[23] Ried-Larsen M, Brønd JC, Brage S, Hansen BH, Grydeland M, Andersen LB, et al. Mechanical and free living comparisons of four generations of the Actigraph activity monitor. Int J Behav Nutr Phys Act. 2012;9:113. PMCID: PMC3463450 doi: 10.1186/1479-5868-9-113. 10.1186/1479-5868-9-113 PMC3463450 22971175

[24] Davis MG, Fox KR, Hillsdon M, Sharp DJ, Coulson JC, Thompson JL (2011). Objectively measured physical activity in a diverse sample of older urban UK adults. Med Sci Sports Exerc.

[25] Orme M, Wijndaele K, Sharp SJ, Westgate K, Ekelund U, Brage S (2014). Combined influence of epoch length, cut-point and bout duration on accelerometry-derived physical activity. Int J Behav Nutr Phys Act.

[26] Hall KS, Howe C a, Rana SR, Martin CL, Morey MC (2013). METs and accelerometry of walking in older adults: Standard versus measured energy cost. Med Sci Sports Exerc.

[27] Ensrud KE, Blackwell TL, Cauley J a, Dam T-TL, Cawthon PM, Schousboe JT (2014). Objective Measures of Activity Level and Mortality in Older Men. J Am Geriatr Soc.

[28] Day N, Oakes S, Luben R, Khaw KT, Bingham S, Welch A (1999). EPIC-Norfolk: study design and characteristics of the cohort. European Prospective Investigation of Cancer. Br J Cancer.

[29] Corder K, Brage S, Ramachandran A, Snehalatha C, Wareham N, Ekelund U (2007). Comparison of two Actigraph models for assessing free-living physical activity in Indian adolescents. J Sports Sci.

[30] Bassett DR, Pucher J, Buehler R, Thompson DL, Crouter SE (2008). Walking, cycling, and obesity rates in Europe, North America, and Australia. J Phys Act Health.

