# Supplementary figures and images for: The descriptive epidemiology of accelerometer-measured physical activity in older adults
Source: Int J Behav Nutr Phys Act. 2016 Jan 7;13:2. doi: 10.1186/s12966-015-0316-z (PMC4704380; doi:10.1186/s12966-015-0316-z)

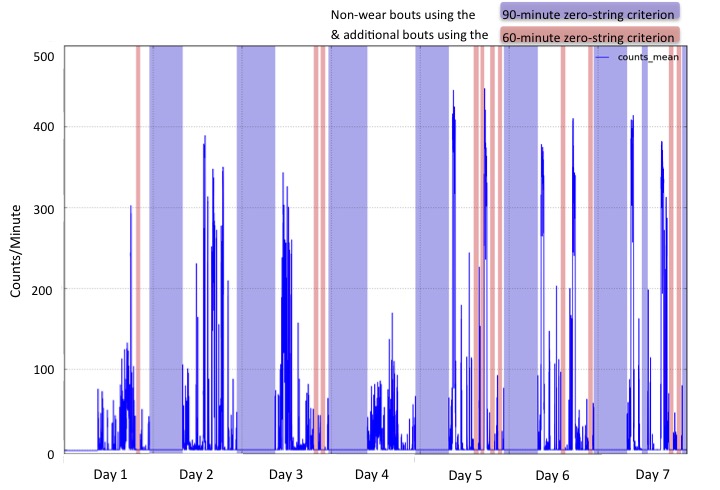

Supplement: Additional file 1: Figure S1. — Example accelerometer file showing time segments classified as non-wear using the 90-min and the 60-min zero string criterions. (JPG 80 kb) [file 12966_2015_316_MOESM1_ESM.jpg]
